# Supplementary figures and images for: A Single-Cell Landscape of Spermioteleosis in Mice and Pigs
Source: Cells. 2024 Mar 22;13(7):563. doi: 10.3390/cells13070563 (PMC11011153; doi:10.3390/cells13070563)

Figure S1

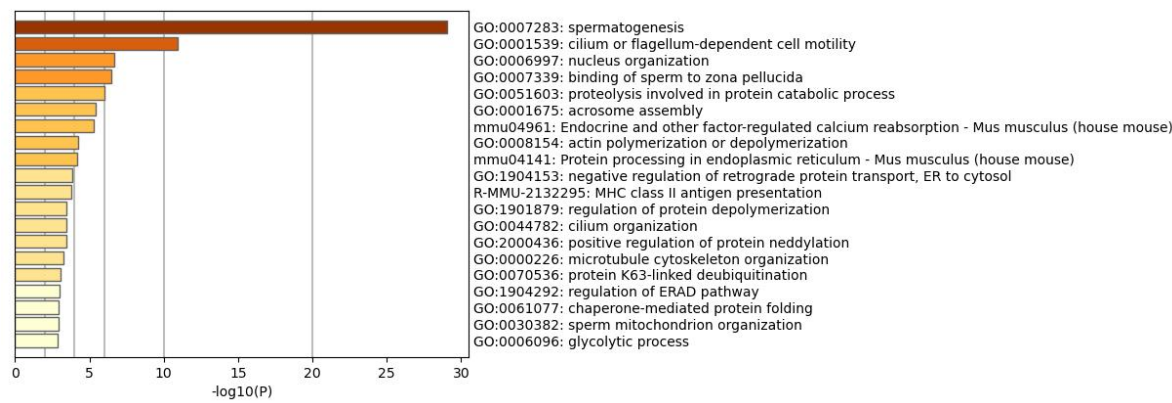

Supplement: Supplementary file 1 [file cells-13-00563-s001.zip › File 2 Supplement Figures/supplement figure S1.pdf]

Figure S2

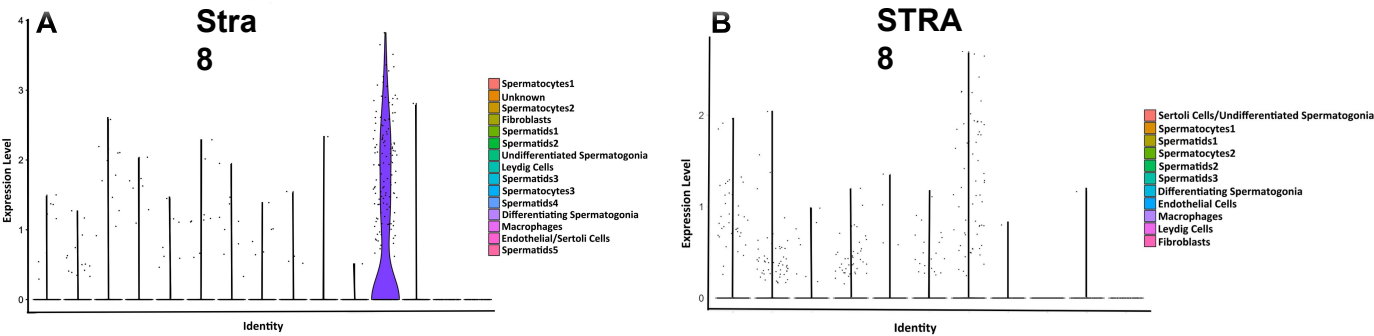

Supplement: Supplementary file 1 [file cells-13-00563-s001.zip › File 2 Supplement Figures/supplement figure S2.pdf]

Figure S3

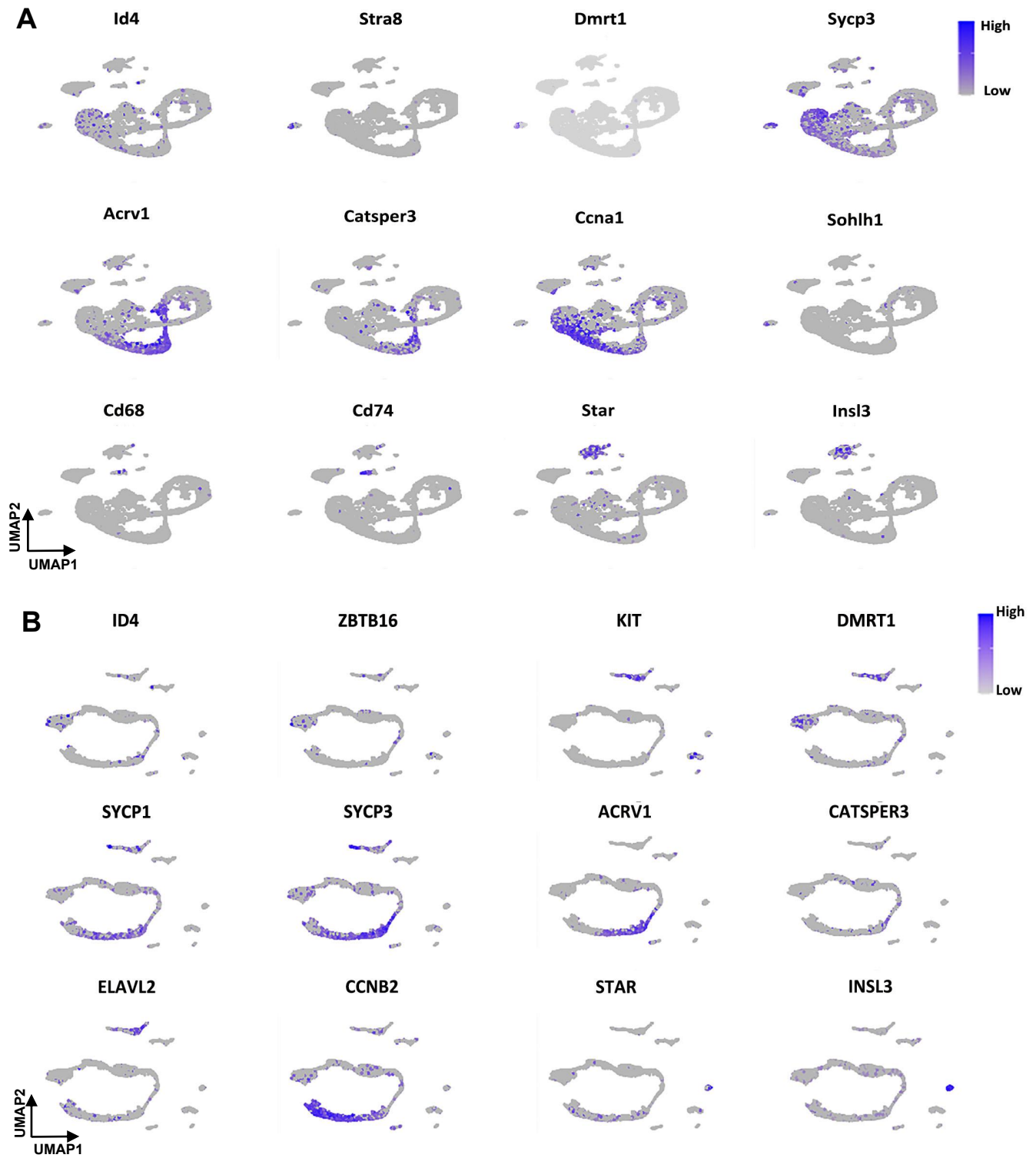

Supplement: Supplementary file 1 [file cells-13-00563-s001.zip › File 2 Supplement Figures/supplement figure S3.pdf]

Figure S4

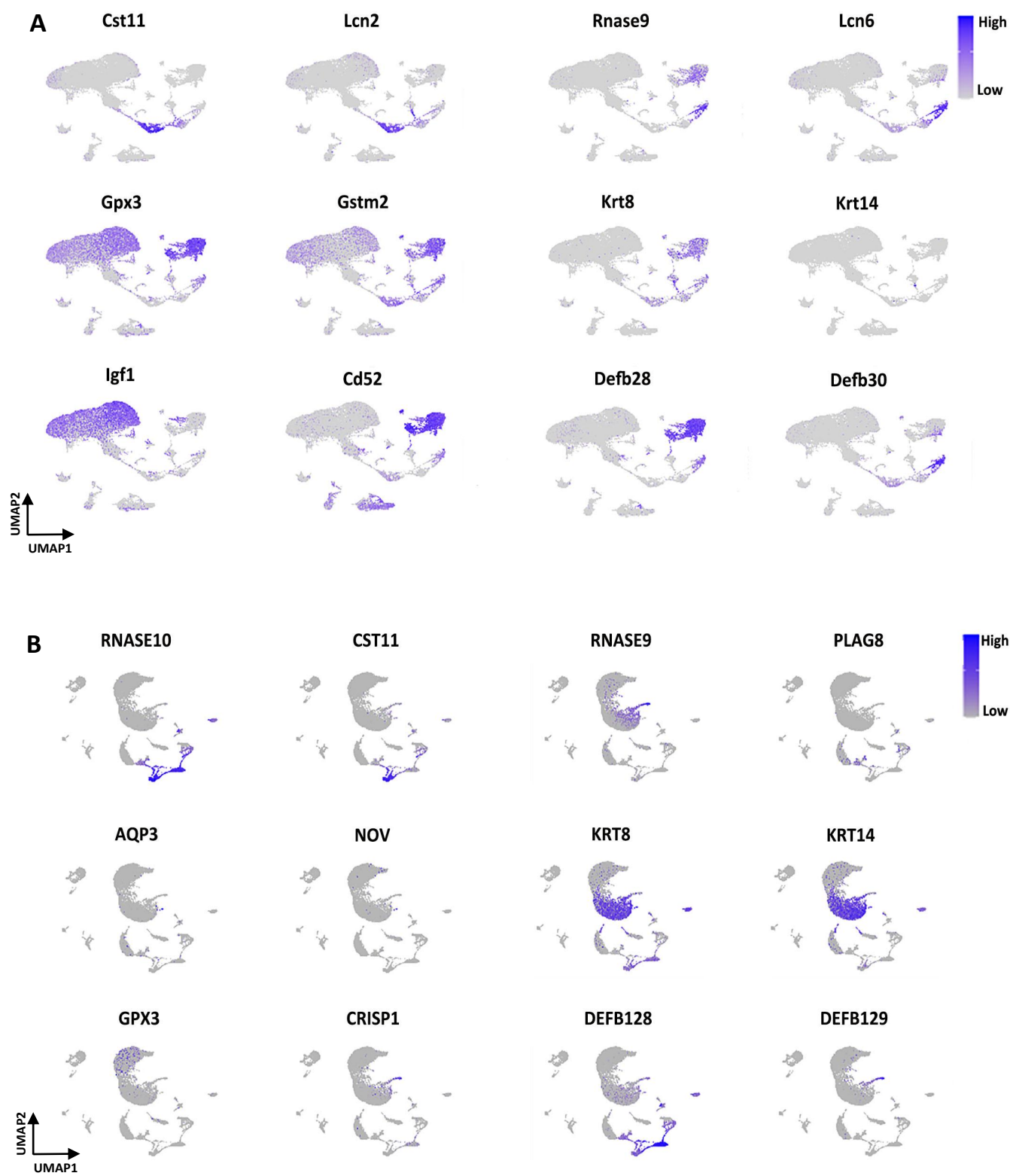

Supplement: Supplementary file 1 [file cells-13-00563-s001.zip › File 2 Supplement Figures/supplement figure S4.pdf]

Figure S5

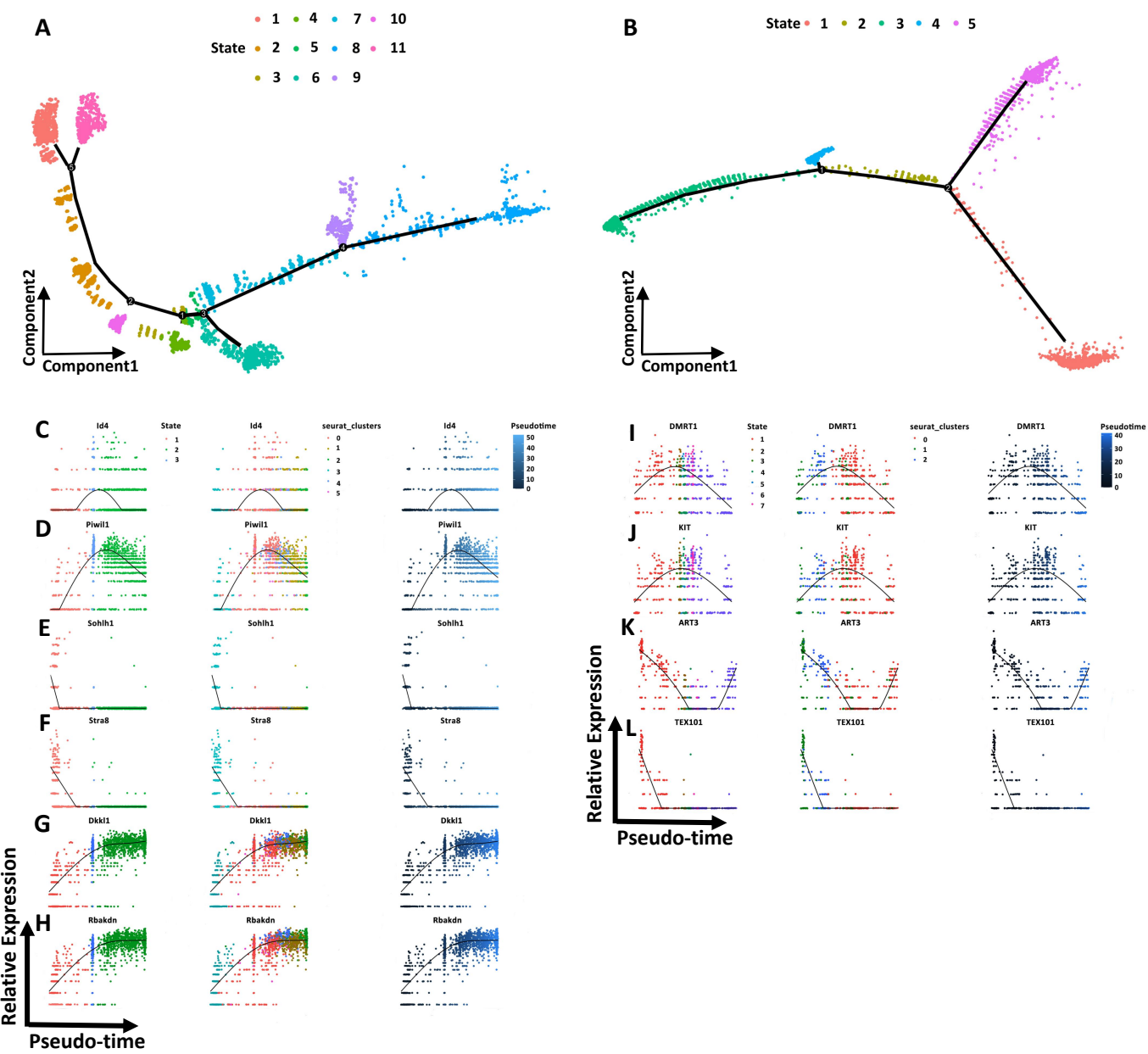

Supplement: Supplementary file 1 [file cells-13-00563-s001.zip › File 2 Supplement Figures/supplement figure S5.pdf]

Figure S6

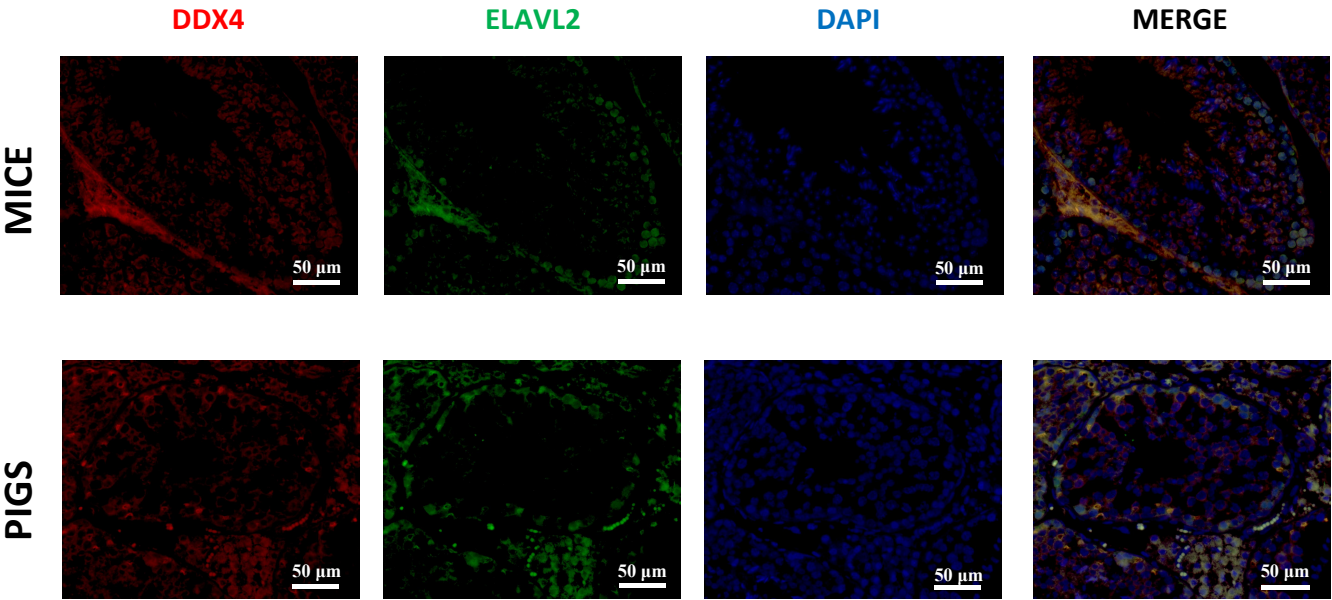

Supplement: Supplementary file 1 [file cells-13-00563-s001.zip › File 2 Supplement Figures/supplement figure S6.pdf]

Figure S7

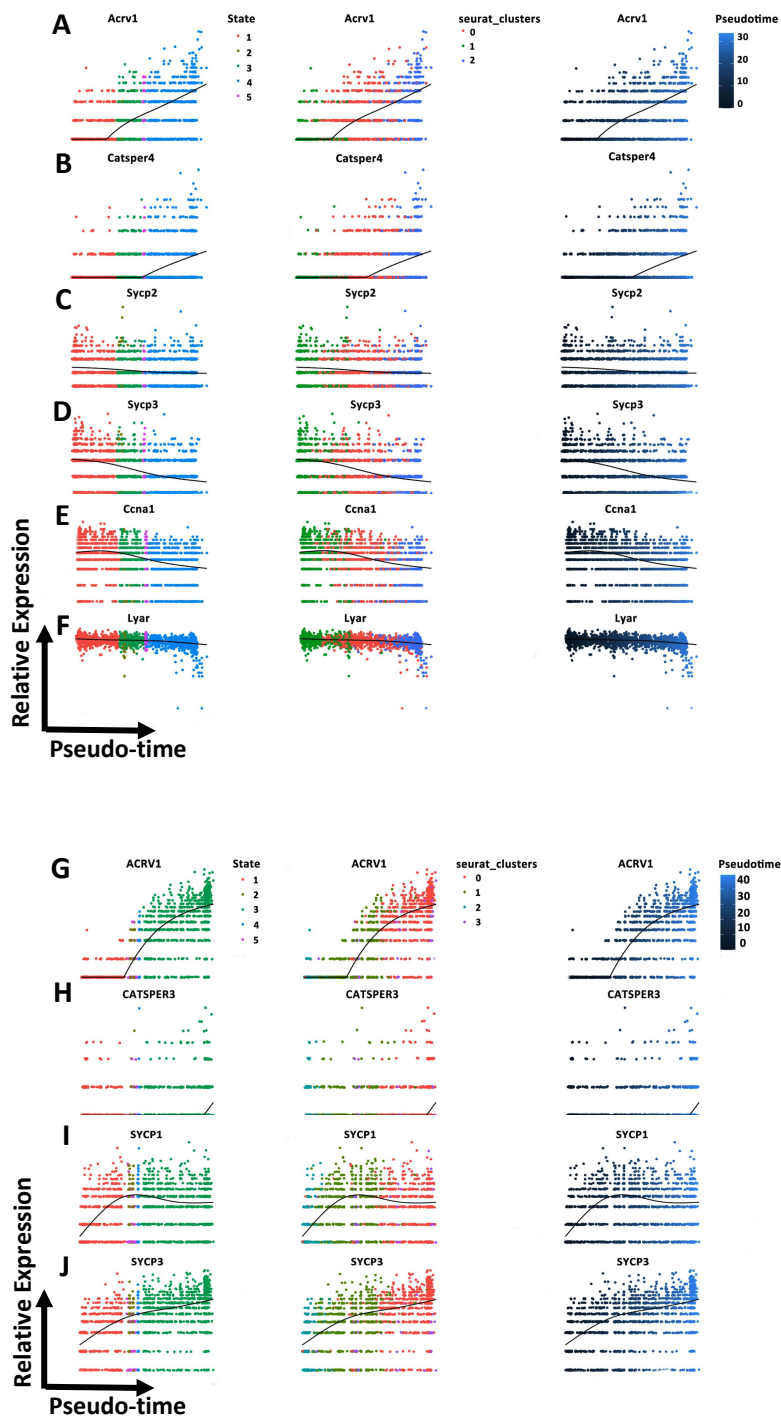

Supplement: Supplementary file 1 [file cells-13-00563-s001.zip › File 2 Supplement Figures/supplement figure S7.pdf]

Figure S8

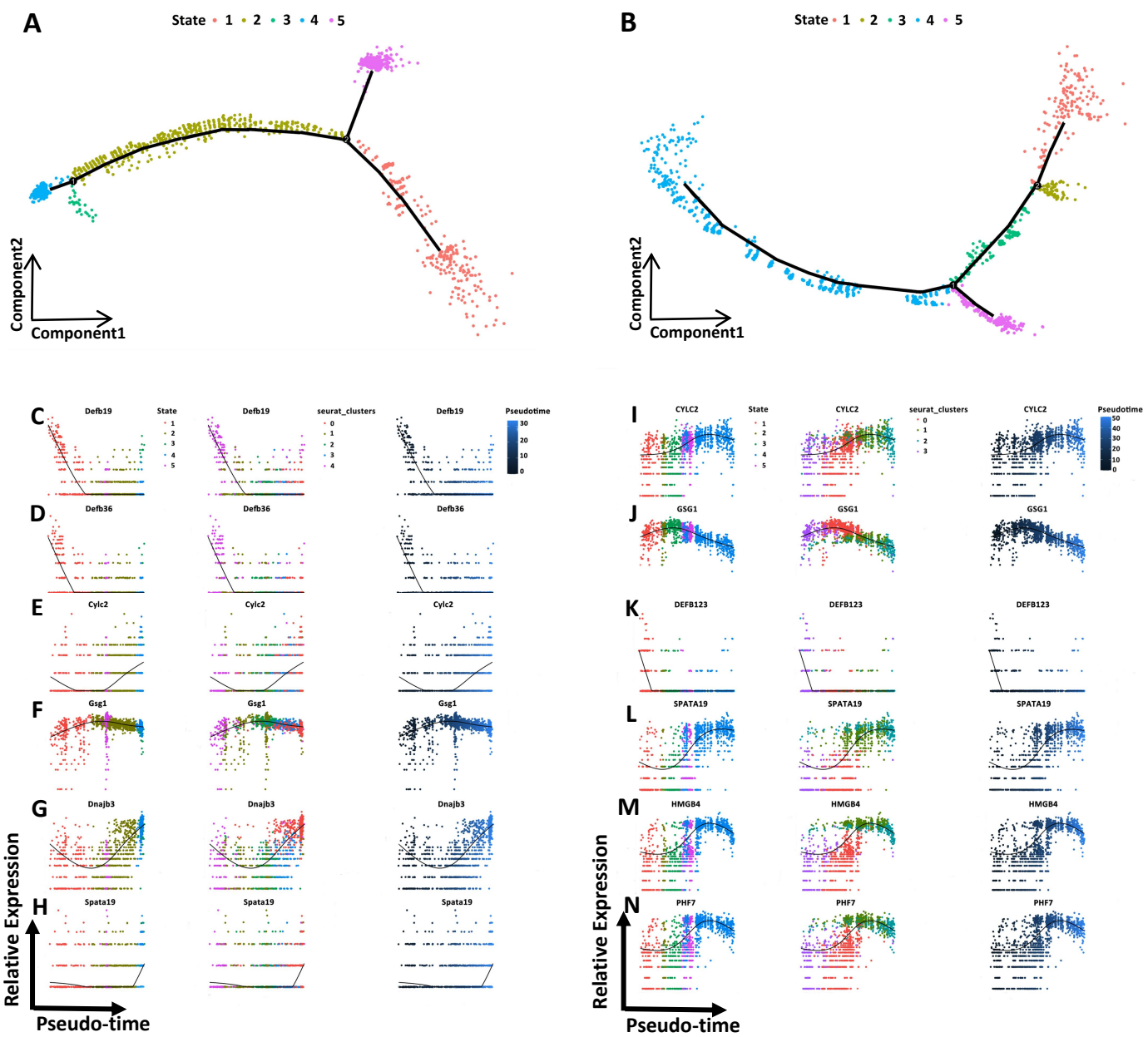

Supplement: Supplementary file 1 [file cells-13-00563-s001.zip › File 2 Supplement Figures/supplement figure S8.pdf]
